# Label-Free Electrochemical Biosensor Platforms for Cancer Diagnosis: Recent Achievements and Challenges

**DOI:** 10.3390/bios13030333

**Published:** 2023-03-01

**Authors:** Vildan Sanko, Filiz Kuralay

**Affiliations:** 1 Department of Chemistry, Gebze Technical University, 41400 Kocaeli, Turkey; 2 Department of Chemistry, Faculty of Science, Hacettepe University, 06800 Ankara, Turkey

**Keywords:** label-free electrochemical detection, electrochemical sensor, cancer diagnosis

## Abstract

With its fatal effects, cancer is still one of the most important diseases of today’s world. The underlying fact behind this scenario is most probably due to its late diagnosis. That is why the necessity for the detection of different cancer types is obvious. Cancer studies including cancer diagnosis and therapy have been one of the most laborious tasks. Since its early detection significantly affects the following therapy steps, cancer diagnosis is very important. Despite researchers’ best efforts, the accurate and rapid diagnosis of cancer is still challenging and difficult to investigate. It is known that electrochemical techniques have been successfully adapted into the cancer diagnosis field. Electrochemical sensor platforms that are brought together with the excellent selectivity of biosensing elements, such as nucleic acids, aptamers or antibodies, have put forth very successful outputs. One of the remarkable achievements of these biomolecule-attached sensors is their lack of need for additional labeling steps, which bring extra burdens such as interference effects or demanding modification protocols. In this review, we aim to outline label-free cancer diagnosis platforms that use electrochemical methods to acquire signals. The classification of the sensing platforms is generally presented according to their recognition element, and the most recent achievements by using these attractive sensing substrates are described in detail. In addition, the current challenges are discussed.

## 1. Introduction

Cancer, which causes premature death in almost all countries of the world, maintains its position at first place even if it is sometimes replaced by cardiac disease. In particular, due to demographic effects and the trends of these effects in cancer incidence in different locations, it is expected that instances of cancer will approximately double in the next 50 years globally. However, cancer does not affect the population of all countries at the same rate, and it is predicted that there will be a higher increase in countries that can be classified as low–middle income [1,2]. The Global Cancer Statistics 2020 report shows that the most common cancer in men is prostate cancer, followed by lung cancer, colorectal cancer and liver cancer, whereas breast cancer and cervical cancer are the most commonly diagnosed cancers in women. In addition, according to the same report, what is striking is that an estimated 19.3 million new cancer cases were detected worldwide and approximately 10.0 million deaths were calculated due to cancer only in 2020 [3].

Regardless of the type, the diagnosis and treatment of cancer at an early stage is very important to reduce both cancer incidence and mortality rates. As the traditional cancer detection method, enzyme-linked immunosorbent assay (ELISA), which detects cancer-specific protein biomarkers and is called the gold standard, is widely known [4]. Also, genomic- and proteomic-based molecular methods such as polymerase chain reaction (PCR), immunohistochemistry (IHC) and radioimmunoassay (RIA) are used for cancer diagnosis [5]. In addition, various clinical tools such as magnetic resonance imaging (MRI), positron emission tomography (PET), endoscopy, sonography, X-ray, computed tomography (CT) and biopsy are extensively utilized [5,6,7]. However, although the mentioned methods and technologies are efficient, most of them are expensive, time-consuming, invasive and limited to the laboratories of some hospitals. Especially with imaging methods, detecting cancer tumors below the millimeter size may be inconclusive. Similarly, invasive methods such as biopsy have the same problems and difficulties in diagnosing early-stage cancer tumors [6].

Early-stage cancer diagnosis increases the survival rate of the patient [8]. In addition, early diagnosis offers several advantages that lead to more appropriate treatment for the patient and even reduce the severity of the cancer [9]. One of the biggest problems limiting early diagnosis in cancer detection is the nonappearance of obvious symptoms in the early stages of cancer; the other is not detecting sufficiently sensitive biomarkers [10]. From an economic point of view, it is known that the costs used for cancer treatment are increasing rapidly, and this cost is expected to increase up to USD 246 billion by 2030. Therefore, detecting cancer at an early stage can reduce the potential economic burden for the patient and society [11]. There is a crucial need to develop low-cost, sensitive, non-invasive (bio)sensors for early-stage cancer diagnosis. In general, biological biomarkers show the genetic characteristics of cancer cells and diagnosis/monitoring of cancer with a biomarker-based biosensor is seen as one of the most promising approaches [12]. These biomarkers can be deoxyribonucleic acid (DNA), ribonucleic acid (RNA), hormones, protein, enzymes and specific cells that can be found in human bodily fluids such as urine, serum, plasma and blood [13,14]. For this aim, electrochemical sensors have been widely used in the field of cancer diagnosis. There are valuable studies in the literature that include various approaches to detect different cancer types such as breast cancer [15], ovarian cancer [16], prostate cancer [17], pancreatic cancer [18] and lung cancer [19]. Electrochemical sensors are prominent tools because they are sensitive, selective, fast, cost-effective, instrumentable and can be performed as on-site analysis [20,21]. Different electrochemical sensing methods such as potentiometric, amperometric, conductometric, impedimetric and voltammetric are used to convert the obtained signal into useful analytical data. Detection methods in biosensors can be grouped as labeled or unlabeled depending on the use of labels as electroactive molecules or nanomaterials. However, labeled systems are complex and expensive as they require an extra labeling process. Conversely, label-free biosensors have shorter analysis time and simplicity and they offer good advantages [22,23,24].

In this review, current label-free cancer diagnosis platforms in the literature, including the last three years, in which the electrochemical method is used as a signal converter, are detailed. Biorecognition elements and mechanisms used in biosensor design for cancer diagnosis are emphasized. In addition, the immobilization method and immobilization matrices, which are important parameters for the activity and stability of a biorecognition element, are also the subject of this study. Finally, current challenges and future perspectives are discussed.

## 2. Electrochemical Techniques as a Sensing Mechanism

Electroanalytical studies are included as a sub-discipline of analytical chemistry, which includes charge transfers in addition to oxidation–reduction reactions [25]. Biorecognition elements, which are one of the parameters that make up the biosensor, are important components for the analyte to be detected. This component needs to be used with a converter so that a meaningful signal can be generated according to the analyte concentration [26]. In an electrochemical transducer system, detectable signals such as current, potential, impedance and conductivity are obtained as a result of the interaction of samples with a bioreceptor. In connection with these signals, electrochemical biosensors are included in various classifications such as amperometric, potentiometric, impedimetric and conductometric. In addition, voltammetric techniques are important and sensitive techniques to help analyte determination [27,28]. Electrochemical detection systems, which provide analytical advantages such as low cost, simple design and portable features, are platforms that can make sensitive and selective detections even in body fluids with complex matrices such as serum [29,30]. Therefore, these detection systems have attracted great attention in biosensor technology owing to their unique properties.

Voltammetric techniques have been commonly utilized. For example, differential pulse voltammetry (DPV), where a pulse is applied to the electrode and provides current measurement. Before the pulse is applied and at the end of each pulse, the current is measured and the difference between the currents is calculated. This procedure effectively reduces the background current due to linear increase, thus resulting in a faradaic current with no capacitive current. The biggest advantage of DPV is a low capacitive current, which leads to high sensitivity. Small steps in DPV also lead to narrower voltammetric peaks, and therefore, DPV is often used to distinguish analytes with similar oxidation potentials. Thus, this technique is preferred in electrochemical cancer biosensors as it exhibits very sensitive properties against the reduction and oxidation of bio-electrochemical species [31,32]. Cyclic voltammetry (CV) is one of the most common methods to obtain information about redox potentials and to investigate the mechanisms and kinetic parameters involved in the reactions of electroactive analytes. In this method, the current between the working and counter electrodes is monitored, but changes in the potential of the working electrode due to the reference electrode are also controlled [33]. In the electrochemical impedance spectroscopy (EIS) technique, the impedance change in both faradaic and non-faradaic modes is measured. As an example, in the measurement system in the faradaic mode, the change in the electron transfer rate caused by the aptamer–analyte interaction is examined. In measurement systems taken in non-faradaic mode, the surface capacitance change due to the aptamer–analyte connection is detected [34]. In the amperometric technique, the working electrode is kept at a constant potential that is sufficient to reduce or oxidize the analyte of interest and the resulting current is monitored over time. Potential selection is critical as only one potential is applied in this technique. Due to the monitoring of current over time at a constant potential, all dynamic changes in the current can be observed [31]. On the other hand, in a potentiometric system based on potential measurements, the principle of changing the potential with the concentration of the analyte is used in the measuring system with the help of a reference electrode with a fixed electrode potential. Besides cancer diagnosis, electrochemical techniques are also highly preferred in routine laboratory analysis and clinical and environmental monitoring analysis [35].

When electrochemical techniques are compared with each other, it is observed that each of them can have limitations in different aspects. For example, the sensitivity of the potentiometric method depending on the environment and temperature is an important limitation. For the limitations of other methods, it can be said that redox elements are needed in the amperometric technique, whereas EIS is sensitive to the environment and requires theoretical stimulation for data analysis [36]. Voltammetric techniques show high selectivity and sensitivity due to the voltammetric peak potential applied to the analyte. However, one of the major problems encountered with these techniques is obtaining overlapping voltammetric responses due to very similar oxidation peak potentials. Various recently developed materials and protocols are used to overcome this problem [37]. Besides this, choosing an appropriate sensing technique for analyte detection can minimize the limitations. Additionally, parameters such as pretreatments applied to the working electrode and the biofunctionality of the electrodes can have a great impact on the precise and effective determination [34].

The electrochemical transformations occurring at the interface of the label-free sensing platform are determined by the affinity between the analyte and the biorecognition elements, regardless of the use of labels [29]. Thanks to the detectable signals obtained by electrochemistry, these techniques are widely preferred not only for cancer detection and follow-up but also for the accurate and sensitive detection of analytes in areas such as the detection of different diseases and environmental and food control [38,39,40,41,42,43]. In an electrochemical biosensor, two different reactions can be observed as a result of the interaction of the electrode surface and the analyte: the first is the positive read signal called “signal-on”, and the other is the negative read signal called “signal-off” [44,45].

Label-free electrochemical biosensors are particularly interesting and important for studies in the biomedical field. In this type of electrochemical biosensor, the information in the reaction is converted into an electrical signal by the direct transfer of electrons between the electrode surface and the biorecognition elements as a result of the interaction between the biomolecule and the analyte [46]. Additionally, the surface characteristics of the electrodes significantly support improving the sensitivity of the biosensor. Therefore, surface modification is also important for good analytical performance. At this point, nanomaterials have been in the scope of scientists. The use of nanomaterials of different sizes, shapes and morphologies together with electrochemical transducers makes it possible to improve properties. Nanowires, metal/metal oxide nanoparticles, carbon nanotubes, graphene or graphene-like structures and conductive nanostructures such as polymers have provided more sensitive biosensors with high surface/volume ratios [47,48]. The scope of this study mainly covers the discussion of the technological developments and also problems/limitations in the development of label-free biosensors containing different biorecognition elements to serve cancer diagnosis.

Despite a lot of effort and good progress in the field of biosensors, it is seen as an inconsistency that only a few of them find a place in the commercial market. The first example of commercial biosensor is the enzymatic glucose biosensor, which is expected to have a market of USD 38 billion by 2027 [49]. This biosensor currently holds approximately 75% of the global biosensor market. There are still outstanding challenges, both to overcome the current constraints and to making the products available commercially. Firstly, understanding the mechanisms of biocatalytic work and charge transfers and also improvements in the properties of biorecognition elements that provide selectivity should be considered. In addition, the use of various nanoparticles and hydrogels has been reported to improve existing deficiencies, although not completely [50,51]. For this purpose, researchers are conducting detailed studies about the effects of parameters on biomolecule (such as enzymes) immobilization and the effect of these parameters on the performance of the biosensor platforms [52]. However, since the biomolecule redox reaction processes are still not fully known, in situ inspection techniques are used for evaluation [53]. Some of the obstacles in the transformation of biosensor studies from laboratory to commercial products are performance and nonspecific surface interaction problems in various body fluids, which have complex matrices [49].

Although electrochemical methods provide several advantages, each method may also have limitations. It is particularly important to focus on and discuss these limitations to put the developed technologies into clinical practice. Reducing or overcoming all the disadvantages could help to develop more accurate and sensitive electrochemical cancer biosensors. More effective platforms for early diagnosis can be created with a multidisciplinary study. In addition, the detection of new cancer biomarkers will greatly benefit the facilitation of early-stage diagnosis and thus the management and control of the cancer disease process. It is expected that the label-free electrochemical methods will increase in reliability after the difficulties we have mentioned have been overcome. As a result, they will find a regular use in the clinical field. To strengthen this reliability, novel and advanced electrochemical cancer biosensors with different perspectives need to be developed.

## 3. Importance of a Label-Free Electrochemical Sensing Platform

A typical electrochemical biosensor is expected to convert signals that are related to the presence of the analyte molecules into measurable quantities with the help of the biorecognition unit. In some cases, various markers/labels or tags are used for the detection of the analyte and the signal is obtained in conjunction with them. These biosensor systems are called label-based biosensors. The use of these labels, which are commonly classified as radioactive-, fluorescent- or chemiluminescence-based, is time consuming and laborious because it requires an extra process. More importantly, it is thought that in this case, the affinity between the biorecognition element and the analyte may be adversely affected. To eliminate these limiting factors, unlabeled detection systems have become highly preferred in recent years. If a direct measurement is made with the biorecognition system, this is called a label-free biosensor system [54].

In a typical label-free biosensor design, sensing can be performed by converting it to optical [55], mechanical [56] or electrical [57] signals and more accurate information can be provided as biorecognition systems are directly used. Within this classification, electrochemical label-free biosensors can be used actively in the field and can be also implanted in the body to detect biological analytes, increasing their future potential [58]. Various electrodes with different biorecognition elements and composite designs have been developed for analytes such as gliotoxin [59], microRNA (miRNA) [60], bacterial pathogens [61] and aflatoxin-B1 [62] in this biosensor group, which combines the advantages of both the electrochemical method and the label-free platform. For the continuation of the remarkable progress of the mentioned electrochemical label-free biosensors, a better understanding of the current working processes is required for the creation of sensitive and selective biosensing systems that find application in wider use. Based on this idea, we have detailed and discussed cancer studies classified on different biorecognition elements.

## 4. Biorecognition Elements for Label-Free Electrochemical Cancer Diagnosis

**Figure 1:**
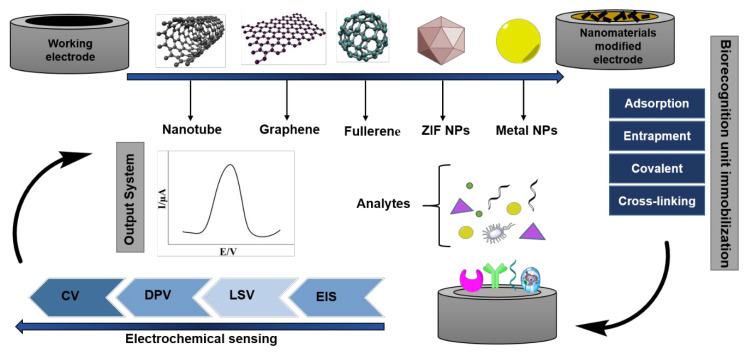
Label-free electrochemical cancer biosensors: electrode modifications such as nanotechnology-based materials, biorecognition immobilization protocols and some of the powerful electrochemical detection techniques.

Basically, antibodies, aptamers, nucleic acids and cells are immobilized to surfaces/interfaces to achieve affinity and selective biorecognition. In this part, the classification of the label-free electrochemical cancer detection systems is divided into categories according to the type of the biorecognition element. Besides this classification, electrode material and the detection technique are also highlighted. Figure 1 demonstrates the schematic presentation of the label-free electrochemical cancer biosensors with successful electrode modifications, such as nanotechnology-based materials, biorecognition immobilization protocols and some of the powerful electrochemical detection techniques.

### 4.1. Nucleic-Acid-Based Label-Free Cancer Biosensors

Nucleic acids are natural biopolymers that store genetic information in humans and almost all organisms [63]. Nucleic acids include DNA and RNA, which are composed of nucleotides. The well-known specific hybridization feature between nucleic acid chains also constitutes the main detection principle of DNA biosensors [64]. The development of biosensors for the detection of DNA sequences is important because of its application in gene identification, molecular diagnosis and drug screening [65]. Nucleic acids can be affected by environmental conditions such as temperature and pH [66]. Nevertheless, in many studies electrochemical signal amplification by means of nucleic acids has been successfully developed for cancer applications [67,68].

Studies in recent years show that excessive secretion of microRNAs is associated with malignancies that cause cancer [15,69,70,71]. In one study, Zhao et al. proposed MXene-molybdenum disulfide (MoS_2_) constructs with thionine and gold nanoparticles for the label-free electrochemical detection of microRNA-21, which plays an important role in the emergence of cancer associated with proliferation/differentiation in cells. The modification of the prepared nanocomposite on glassy carbon electrode (GCE) was performed by drop casting. Then, the hairpin capture probe was dropped onto the modified electrode. The hybridization event was carried out in the presence of the target and a hairpin probe 2. The detection method was square wave voltammetry (SWV). Thanks to this structure, the capture probe immobilization was improved, the amplification of the electrochemical signal was achieved and microRNA-21 detection in the linear measurement range of 100 fM to 100 nM was obtained with a detection limit of 2 fM [72].

Pothipr et al. described a gold nanoparticle-dye/poly(3-aminobenzylamine)/two-dimensional molybdenum selenide (MoSe_2_)-based electrochemical label-free biosensor for breast cancer diagnosis that could detect cancer antigen 15-3 and microRNA-21 simultaneously. Based on the complexity of the immune system in the human body and therefore the inadequacy of cancer assays using single biomarker systems, they introduced this bidirectional detection platform produced on a two-screen printed carbon electrode. DPV was used for the evaluation of the electrochemical performance of the biosensor and the detection limit was found to be 1.2 fM for microRNA-21 detection [73]. Jafari-Kashi et al. presented a DNA biosensor for the detection of cytokeratin 19 fragment 21-1, which is associated with lung cancer. They preferred DPV as an electrochemical technique to examine the interaction between the capture probe and target using GCE modified with reduced graphene oxide, polypyrrole, silver nanoparticles and single-stranded DNA (ssDNA). With this technique, no peak was detected before DNA hybridization, but a distinctive peak was obtained after hybridization according to the oxidation of guanine. They declared that the label-free DNA biosensor showed a good result for detection of cytokeratin 19 fragment 21-1, with a wide linear measurement range and a 2.14 fM limit of detection [74]. Avelino et al. presented a polypyrrole film containing DNA immobilized chitosan/zinc oxide nanoparticles for the diagnosis of myelocytic leukemia by BCR/ABL fusion gene detection. Oxidation and reduction steps were observed in line with the voltammetric measurements taken in 10 mM [Fe(CN)_6_]^3–/4–^. It is also stated that the biosensor was designed as a result of bioactivity tests and could be used as a new biosensing platform that enabled the identification of early-stage cancer [75].

### 4.2. Aptamer-Based Label-Free Cancer Biosensors

Aptamers are single-stranded DNA or RNA molecules that can usually be synthesized using an in vitro method. In fact, RNA-based aptamers were first found in 1990, followed by DNA-based aptamers, with the development of in vitro selection/amplification for the isolation of RNA sequences that could specifically bind to molecules [76]. In aptamer-based electrochemical sensors, it is necessary to be able to detect the conformational changes caused by the presence of the aptamer on the electrode surface for obtaining a signal [77]. Aptamers are widely used in the development of biosensors due to their high specificity, easy synthesis, simple modification and high chemical stability [78]. They offer the advantages of more cost-effective production, easy modification and thermal stability, especially when compared with monoclonal antibodies. After the aptamers are immobilized on a conductive matrix, their redox-active moieties allow the formation of aptamer–target complexes and thus the design of various electrochemical biosensors with the realized electron transfer properties [76]. The most important problem in this electrochemical process can be the generation of a determinable signal between the target analyte and the aptamer. In order to solve this problem, electrochemically active labeling units such as hemin [79], ferrocene [80] and methylene blue [81] have been introduced. However, labeling of aptamers introduces known disadvantages such as time consumption, poor affinity performance and cost [82].

In recent years, aptamers have attracted great interest in electrochemical label-free biosensor design, which has applications in the diagnosis and follow-up of various cancers. Label-free aptasensors also require an increased surface area to improve weak signal intensity. Nanomaterials contribute greatly to increasing the surface area because they act as electron-transfer tunnels, which increase the electrical communication between the redox regions of the aptamer and the electrode surface [83]. Zhang et al. developed a label-free aptasensor for the detection of cancer antigen 125 by immobilizing aptamer on the surface of nickel hexacyanoferrate nanocubes/polydopamine functionalized graphene. DPV was utilized for electroanalytical studies in this work, which was designed to provide a detectable electrochemical response with the help of increasing surface area and conductivity. Thanks to the insulating structure formed as a result of the combination of aptamer and cancer antigen 125 (CA125), or in other words aptamer–CA125 complex, the peak current value decreased as the analyte concentration increased. The linear measurement range and limit of detection were calculated as 0.10 pg mL^−1^–1.0 μg mL^−1^ and 0.076 pg mL^−1^, respectively. The measurements were carried out in phosphate buffer solution (PBS) [82]. In another study, a paper-based electrochemical label-free aptasensor was fabricated for the detection of epidermal growth factor receptors. Interestingly, the concept of origami as a valve for a paper-based biosensor was used in this study. As a result of the biochemical reaction, the data became an electrochemical response with the presence of the nanocomposites containing amino functionalized graphene/thionine/gold. This system in the form of origami was designed to increase the penetration of the liquid and shorten the time taken for flow, resulting in a shorter test time. The linear concentration range obtained with the sensor was from 0.05 ng mL^−1^ to 200 ng mL^−1^ and it had a detection limit of 5 pg mL^−1^ [84].

### 4.3. Antibody-Based Label-Free Cancer Biosensors

Antibodies are protective proteins produced by the immune system in response to the presence of antigens, including pathogens and toxic materials [78]. Biosensors that offer the advantages of high binding affinity and specificity and use antibodies for biorecognition take the advantage of the high affinity between antibodies and antigens for detection and are called immunosensors [85,86]. However, there are some parameters that limit their use. Apart from being adversely affected by environmental conditions and having difficulties for storage, it can be said that the production of polyclonal antibodies in animals is difficult and costly. Moreover, polyclonal antibodies may lack selectivity as they can have affinity for different epitopes [87]. With the help of the new and improved sensor interfaces developed in recent years, some disadvantages have been overcome and many antibody-based sensitive and selective label-free electrochemical biosensors have been designed. Also, these limitations pave the way for the development of new forms of biorecognition units that can replace antibodies, thus introducing new biosensor projections to the field.

Various electrochemical techniques have been used for antibody-based biosensors for gastric cancer [88], breast cancer [89,90,91,92], ovarian cancer [93,94,95,96], bladder cancer [97], colorectal cancer [98], lung cancer [99], prostate cancer [100,101,102,103,104,105], liver cancer [106] and more. In a study for a label-free electrochemical immunosensor developed for early-stage detection of prostate cancer, the surface of the indium tin oxide electrode was firstly coated with chitosan and reduced graphene oxide, and then the specific polyclonal anti-prostate-specific antigen (PSA) antibody as a recognition element was immobilized on the surface. It was determined that a linear decrease had been observed in the peak current values of the redox probe by using DPV with increasing concentrations of the antigen. It is reported that the linear measurement range determined for prostate-specific antigen detection was between 1 pg mL^−1^ and 5 ng mL^−1^, and the limit of detection was 0.8 pg mL^−1^ [107].

CA125 was detected by DPV using a layer-by-layer assembly of ordered mesoporous carbon, gold nanoparticles and MgAl-layered double hydroxides containing ferrocene carboxylic acid composite. It is explained that the conductivity increased significantly with the addition of the ferrocene component to the composite. The electrochemical performance of the biosensor was determined based on the change of the peak current observed in the voltammogram at +0.27 V according to the ferrocene in the presence of different CA125 antigen concentrations. It is stated that the peak current value obtained with the increase in the CA125 concentration changed inversely, since the complex formed between the antigen and the antibody. The linear measuring range and limit of detection of the biosensor were described as 0.01 U mL^−1^–1000 U mL^−1^ and 0.004 U mL^−1^, respectively [108]. A label-free sandwich type biosensor was developed for the electrochemical detection of cytokeratin fragment antigen 21-1 (CYFRA 21-1), a lung cancer biomarker. An antibody–antigen–antibody sandwich structure was formed between the 4-(2-trimethylsilylethinyl)benzoic acid gold electrode used as a bridge and the poly(ε-caprolactone)-b-poly(ethylene oxide) copolymer. The linear concentration range and limit of detection for the sensor determined by electrochemical impedance spectroscopy were declared as 1.0 pg mL^−1^ to 10 ng mL^−1^ and 0.125 pg mL^−1^, respectively. According to the impedance results, the electrochemical responses showed a linear response with the concentration of CYFRA 21-1 [109].

Liu et al. developed a gold nanoparticle/polyethyleneimine/reduced graphene oxide nanocomposite for the electrochemical detection of matrix metalloproteinase-1, a cancer biomarker, based on the knowledge that gold nanoparticles were supportive in maintaining the reversibility of redox reactions in electroanalytical reactions. They determined that the biosensor performance obtained by DPV had an operating range of 1 ng mL^−1^ to 50 ng mL^−1^. In this work, the peak current value obtained from voltammetry decreased due to the increased antigen concentration blocking on the electrode surface. In the electrochemical measurements taken in 5 mM Fe(CN)_6_^3−/4−^ medium, it is stated that an insulating layer was formed due to the antigen–antibody complex, and therefore, a repulsive electrostatic interaction occurred between the antigen and Fe(CN)_6_^3−/4−^ [110]. Zhu et al. also developed a carbon-based nanocomposite to take advantage of its high surface area and good conductivity properties. The surface was used for the construction of an immunosensor for the detection of alpha-fetoprotein, which is a liver cancer biomarker. They calculated a linear measurement range of 0.10 ng mL^−1^ to 420 ng mL^−1^ and a limit of detection of 0.03 ng mL^−1^ using square wave voltammetry, a method that could suppress background current and provide sensitivity to the biosensor system [106].

### 4.4. Cell-Based Label-Free Cancer Biosensors

The use of cells as a biorecognition element dates back to the early 1970s and it is still preferred today. Cells offer an interesting alternative to other biorecognition units such as antibodies, enzymes and nucleic acids thanks to their relatively easy production and lower cost than antibodies and purified enzymes. As an example, since whole cells offer a multi-enzyme alternative, they can be preferred in the development of biosensors for the simultaneous determination of various analytes. In addition, cell-based biosensors enable in situ monitoring using suitable substrates [78,111,112]. However, some limitations such as maintenance and immobilization of cells can arise [113].

Human cervical carcinoma (HeLa) cells were used as a biorecognition unit in an electrochemical label-free cytosensor to evaluate the anticancer activity of pinoresinol, which had biological properties such as anticancer, anti-inflammatory and antifungal effects. HeLa cells were immobilized on a GCE surface modified with multi-walled carbon nanotubes and gold nanoparticles, and the performance of the biosensor was evaluated by electrochemical impedance spectroscopy with different pinoresinol concentrations. The limit of detection value for the biosensor, which showed a linear correlation with the pinoresinol concentration range of 10^2^ to 10^6^ cells mL^−1^, was reported as 10^2^ cells mL^−1^ [114]. Another cell-based label-free electrochemical biosensor was developed to investigate the interactions of cancer cells (HepG2 cells and A549 cells) with molecules and to screen anticancer drugs. Cancer cells were immobilized on the GCE coated with N-doped graphene–Pt nanoparticles–chitosan and polyaniline. It is stated that this electrode surface might be suitable for examining different cell lines by changing the targeted cells as a result of the electrochemical properties examined by DPV with its large surface area and catalytic properties [115].

Liu et al. carried out the detection of cell surface glycan that played an important role in processes such as cancer cell metastasis by means of a nano channel ion channel of porous anodic alumina hybrid combined with an electrochemical detector. Thus, the enhanced ionic current caused by the array nano channels along with the ionic current rectification gave a precise current response. The alumina was functionalized with aminopropyltriethoxysilane and glutaraldehyde to immobilize the cell surface glycan. The linear working range was obtained from 10 fM to 10 nM, and the limit of detection was calculated to be approximately 10.0 aM. It is stated that this biosensor was a promising alternative that could be used in cancer diagnosis and an important platform for label-free detection of cell surface glycan [116].

Despite the advantages of cell-based electrochemical biosensors, there are also various disadvantages faced by designers such as reproducibility and inability to selectively place cells at detection sites [117]. In addition, some difficulties in terms of electrochemical techniques such as amperometric and impedimetric have been reported in the literature. For example, the difficulties often observed in electrochemical impedance spectroscopy-based studies are that the measured electrochemical response is the total change produced by a set of cells and poor selectivity. Emerging technology, nanomaterial selection, new immobilization matrices, integration of different transducer mechanisms and advances in the control of the sensor interface are some of the promising approaches to overcome these challenges [105,106].

## 5. Immobilization Strategies of Biorecognition Elements

**Figure 2:**
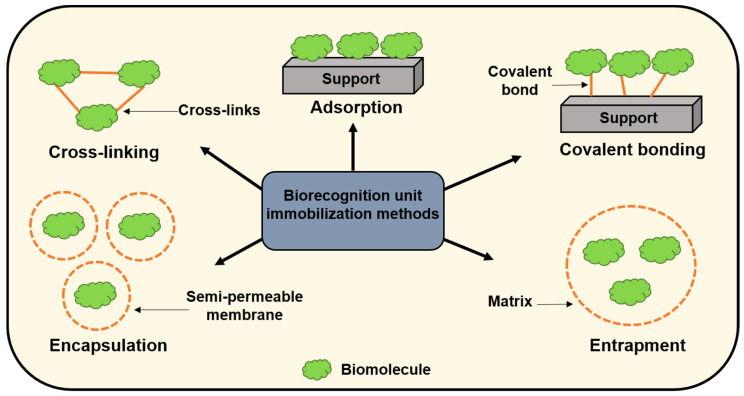
Various immobilization methods for the biorecognition elements.

Biorecognition element immobilization or its integration is one of the important processes to be considered, since this step thoroughly affects the analytical performance of all types of biosensors. The efficient immobilization of the biorecognition element is a process applied to overcome the problems such as loss of activity and stability by integrating biomolecules into a suitable support material. The immobilization methods are classified as adsorption, covalent bonding, cross-linking, etc., according to the type of the biomolecule to be immobilized and the structure of the immobilization surface [118]. These methods are illustrated in Figure 2.

In Table 1, the immobilization methods used by some of the studies within the scope of this review are indicated. Some cancer detection studies in the literature for recent years, different biorecognition units, other biosensor components and the parameters used in these studies are listed. Metals, metal oxides, conductive polymers, biopolymers, carbon-based structures, quantum dots and their composites [93,100,107,109,119,120] have been used as the immobilization matrices for label-free electrochemical cancer biosensors. In general, electrostatic interactions can have negative effects on the stability of the biorecognition element or the repeatability of the biosensor [121,122]. However, these methods, which have very simple processes, are still actively used in the surface immobilization of many electrodes. The entrapment method also offers specific properties and contributes to the improvement of chemical and thermal stability. However, leakage and low biological activity limit this method. To overcome the leakage problem, crosslinkers are preferred in the immobilization step. However, at this stage, excessive chemical requirements are necessary [123].

In the study of Yaiwong et al., an immunosensor for label-free electrochemical cancer detection was developed. Electrostatic interaction was carried out for the immobilization of the anti-metalloproteinase-7 (MMP-7) capture antibody, which was used as a biorecognition element, on the surface of the screen-printed carbon electrode (SPCE) coated with two-dimensional (2D) MoS_2_/graphene oxide [124]. More commonly, immobilization methods by covalent or cross-linking over carboxyl or amine groups are robust and reproducible ways to obtain an effective biosensor interface. Glutaraldehyde or carbodiimide structures that act as bridges in these binding reactions are preferred [121]. As an example, Yan et al. coated the surface of an indium tin oxide electrode with chitosan-modified reduced graphene oxide nanocomposite for prostate cancer detection. In order to detect prostate-specific antigens with this biosensor, they immobilized the recognition antibodies onto the electrode surface by covalent bonding. Chitosan naturally provided a large number of amino groups to the electrode surface, and glutaraldehyde, a bifunctional bridge, was used for covalent immobilization of the anti-PSA antibody with amino groups. Thus, a label-free electrochemical immunosensing platform based on antibody–antigen affinity was developed [107].

Echeverri et al. immobilized the anti-β-1,4-galactosyltransferase-V (β-1,4-GalT-V) antibody biorecognition element on the self-assembled monolayer (SAM)-coated SPCE by covalent bonding for the detection of colorectal cancer. The SAM provided a carboxylic acid group that allowed for antibody binding [98]. Generally, N-(3-dimethylaminopropyl)-N′-ethylcarbodiimide hydrochloride (EDC) and N-hydroxysuccinimide (NHS) pairs are used for this type of covalent bonding. In this way, a bridge is formed between the amine and carboxyl groups and a high binding efficiency is achieved [121]. Although covalent bonding seems to offer good efficiency and is an advantageous method, it can also have various disadvantages in some cases. For example, denaturation may occur due to the undesirable site orientation of the biorecognition element, and in addition, the bridging compounds are needed to use in the covalent bonding reaction. Therefore, there can be a decrease or disappearance of the biocatalytic effect expected from the biorecognition unit [125]. Moreover, covalent bonding, which causes a tight binding, can also restrict the movement of the biorecognition elements, which may also cause a loss of activity [126].

**Table 1:** Electrochemical-based label-free biosensors for cancer detection: biorecognition elements, sensor platforms, immobilization methods and electroanalytical performances.

Biorecognition Elements	Other Components	Immobilization Method	Cancer Type	Analyte	Electrochemical Technique	Limit of Detection	Linear Range	References
Biotinylated DNA probe	Multi-walled carbon nanotubes non-covalently functionalized with avidin	Covalent binding	Breast cancer	BRCA1	Electrochemical impedance spectroscopy	330 aM	1.0 fM–10 nM	[67]
Three-dimensional (3D) DNA walker	Au nanoparticles/encapsulation of glucose oxidase in zeolitic imidazolate framework-8 (ZIF-8)	Electrostatic adsorption	Cancer	MicroRNA	Differential pulse voltammetry	29 pM	0.1 nM–10 μM	[68]
Hairpin probe (H1)	MXene/MoS_2_/thionine/Au nanoparticles	Adsorption	Cancer	MicroRNA-21	Square wave voltammetry	26 fM	100 fM–100 nM	[72]
Anti-CA 15-3 antibodies	Gold nanoparticle-dye/poly(3-aminobenzylamine)/two dimensional MoSe_2_/graphene oxide	Covalent binding	Breast cancer	CA 15-3	Differential pulse voltammetry	0.14 U mL^−1^	0.0–500 U mL	[73]
DNA-21	Gold nanoparticle-dye/poly(3-aminobenzylamine)/two dimensional MoSe_2_/graphene oxide	Adsorption	Breast cancer	MicroRNA-21	Differential pulse voltammetry	1.2 fM	0.0–1000 pM	[73]
DNA	Reduced-graphene oxide/polypyrrole/silver nanoparticles	Covalent binding	Lung Cancer	Cytokeratin 19 fragment 21–1 (CYFRA21-1)	Differential pulse voltammetry	2.4 fM	1.0 × 10^−14^–1.0 × 10^−6^ M	[74]
DNA-aptamer probe	α-Fe_2_O_3_/Fe_3_O_4_@Au	Adsorption	Ovarian cancer	CA125	Differential pulse voltammetry	2.99 U mL^−1^	5–125 U mL^−1^	[77]
Aminoated CA 125 aptamers	Nickel hexacyanoferrate nanocubes/polydopamine functionalized graphene	Covalent binding	Ovarian cancer	CA125	Differential pulse voltammetry	0.076 pg mL^−1^	0.10 pg mL^−1^–1.0 μg mL^−1^	[82]
Aptamer	Graphene oxide functionalized with aspartic acid	Covalent binding	Cancer	Cytochrome c	Differential pulse voltammetry	0.74 nM	10 nM–100 μM	[83]
Epidermal growth factor receptor (EGFR) aptamers	Amino-functionalized graphene/ thionine/gold particle nanocomposites	Covalent binding	Cancer	EGFR	Differential pulse voltammetry	5 pg mL^−1^	0.05–200 ng mL^−1^	[84]
CA72-4 antibodies	Carbon nanotube–graphene oxide hybrid	Covalent binding	Gastric cancer	Antigen 72-4	Differential pulse voltammetry	0.4 U mL^−1^	2.0–80.0 U mL^−1^	[88]
Calreticulin antibody	Electrodeposited single-walled carbon nanotubes and polymerized oxiran-2-yl methyl 3-(1H-pyrrol-1-yl) propanoate monomer	Covalent binding	Breast cancer	Calreticulin	Electrochemical impedance spectroscopy	0.0046 pg mL^−1^	0.015–60 pg mL^−1^	[89]
Cancer antigen (CA 15-3) antibody	Ternary silver/titanium dioxide/reduced graphene oxides nanocomposite	Covalent binding	Breast cancer	CA 15-3 antigen	Amperometry	0.07 U mL^- 1^	0.1–300 U mL^−1^	[90]
Anti-cancer antigen (CA125) antibody	Boron nitride nanosheets	Physical adsorption	Ovarian cancer	CA125	Differential pulse voltammetry	1.18 U mL^−1^	5–100 U mL^−1^	[94]
Anti-nuclear matrix protein 22 (NMP22)	Reduced graphene oxide/tetraethylene pentamine/Cu-based metal organic frameworks deposited silver nanoparticles	Covalent binding	Bladder cancer	NMP22	Differential pulse voltammetry	33.33 fg mL^−1^	0.1 pg mL^−1^–1000 ng mL^−1^	[97]
Anti-β-1,4-galactosyltransferase-V (β-1,4-GalT-V) antibody	Self-assembled monolayer-coated screen-printed gold electrode	Covalent binding	Colorectal cancer	β-1,4-GalT-V glycoprotein	Electrochemical impedance spectroscopy	7 pM	5–150 pM	[98]
Prostate-specific membrane antibody (PSMA)	Cysteamine-modified gold nanoparticles	Cross-linking	Prostate cancer	PSMA protein	Differential pulse voltammetry	0.47 ng mL^−1^	0–5 ng mL^−1^	[100]
Anti-prostate-specific antigen (PSA)	Chitosan, graphene, ionic liquid and ferrocene cryogel	Chemical adsorption	Prostate cancer	PSA	Differential pulse voltammetry	4.8 × 10^−8^ ng mL^−1^	1.0 × 10^−7^–1.0 × 10^−1^ ng mL^−1^	[103]
Anti-alpha-fetoprotein (AFP)	MnO_2_ functionalized mesoporous carbon hollow sphere	Cross-linking	Liver cancer	AFP	Square wave voltammetry	0.03 ng mL^−1^	0.10–420 ng mL^−1^	[106]
Polyclonal anti-PSA antibody	Chitosan–graphene-modified indium tin oxide electrode	Covalent binding	Prostate cancer	Prostate-specific antigen	Amperometry	0.8 pg mL^−1^	1–5 ng mL^−1^	[107]
Primary antibody (Ab1)	Linear poly(ε-caprolactone)- b-poly(ethylene oxide) copolymer	Cross-linking	Lung cancer	CYFRA 21-1	Electrochemical impedance spectroscopy	0.125 pg mL^−1^	1 pg mL^−1^–10 ng mL^−1^	[109]
Human cervical carcinoma (HeLa) cells	Carboxylated multiwalled carbon nanotubes/gold nanoparticles	Adsorption	Cervical cancer	Pinoresinol	Electrochemical impedance spectroscopy	10^2^ cells mL^−1^	10^2^–10^6^ cells mL^−1^	[114]
Anti-matrix metalloproteinase (MMP)-7 capture antibodies	Two-dimensional molybdenum disulfide/graphene oxide nanocomposite	Electrostatic interactions	Pancreatic and colorectal cancers	MMP-7	Differential pulse voltammetry	0.007 ng mL^−1^	0.010–75 ng mL^−1^	[124]
Antibodies specific to IL-8 (Anti-IL-8)	Silver molybdate nanoparticles	Covalent binding	Oral cancer	IL-8	Differential pulse voltammetry	90 pg mL^−1^	1 fg mL^−1^–40 ng mL^−1^	[127]
Human epidermal growth factor receptor 2 (HER2) antibody	Fe_3_O_4_/TMU-21/multi-walled carbon nanotubes	Cross-linking	Breast cancer	HER2	Amperometry	0.3 pg mL^−1^	1.0 pg mL^−1^–100 ng mL^−1^	[128]
Carcinoembryonic antigen (CEA) antibody	Fe_3_O_4_@Au nanoparticles	Adsorption	Cancer	CEA	Linear sweep voltammetry	0.10 pg mL^−1^	0.001–100 ng mL^−1^	[129]
DNA	Exo-III-assisted target recycling and dual enzymes	Covalent binding	Oral cancer	ORAOV1	Electrochemical impedance spectroscopy	0.019 fM	0.05 fM–20 pM	[130]
Capture strand	Au nanoparticles	Adsorption	Brain cancers	Cerebrospinal fluid microRNAs	Differential pulse voltammetry	56 fM	0.5–80 pM	[131]
CEA aptamer (AptGAC- P)	6-Mercapto-1-hexanol (MCH)/cpDNA2/gold	Adsorption	Cancer	CEA	Differential pulse voltammetry	0.24 ng mL^−1^	2–45 ng mL^−1^	[132]
Carcinoembryonic antigen aptamer	Au nanoparticles	Self-assembly	Lung cancer	CEA	Electrochemical impedance spectroscopy	0.085 ng ml^−1^	0.2–15.0 ng ml^−1^	[133]
AS1411 aptamer	Reduced graphene oxide–chitosan–gold nanoparticle	Covalent binding	Breast cancer	MCF-7 cancer cells	Electrochemical impedance spectroscopy	4 cells mL^−1^	1 × 10–1 × 10^6^ cells mL^−1^	[134]
DNA aptamer	Gold electrode	Covalent binding	Cancer	Cluster of differentiation-44 (CD44)	Electrochemical impedance spectroscopy	0.087 ng mL^−1^	0.1–1000 ng mL^−1^	[135]
PDGF-BB affinity aptamers	Carboxyl-functionalized photoresist-derived carbon	Covalent binding	Cancer	Platelet-derived growth factor-BB (PDGF-BB)	Cyclic voltammetry	7 pM	0.01–50 nM	[136]
DNA	Graphene oxide–chitosan/polyvinylpyrrolidone–gold nano urchin	Covalent binding	Lung Cancer	miR-141	Square wave voltammetry	0.94 fM	2.0–5.0 × 10^5^ fM	[137]
DNA-21	Graphene/polypyrrole/gold nanoparticles	Electrostatic interaction	Cancer	miRNA-21	Differential pulse voltammetry	0.020 fM	1.0 fM to 1.0 nM	[138]
DNA	Chitosan-capped gold nanoparticles	Electrostatic interaction	Cervical cancer	HPV-16	Cyclic voltammetry/square wave voltammetry	1.0 pM	1 pM–1 μM	[139]
ssDNA	L-cysteine functionalized ZnS quantum dots	Covalent binding	Ovarian cancer	miR-200a	Electrochemical impedance spectroscopy	8.4 fM	1.0 × 10^−14^–1.0 × 10^−6^ M	[140]
ssDNA	Reduced graphene oxide/polyaniline nanofibers	Electrostatic interaction	Breast cancer	BRCA1	Differential pulse voltammetry	3.01 × 10^−16^ M	1.0 × 10^−15^–1.0 × 10^−7^ M	[141]
acpcPNA-T9 probe	Ag@Au core–shell nanoparticles electrodeposited on graphene quantum dots	Adsorption	Cancer	miRNA-21	Chronoamperometry	5 pM	5 pM–5 mM	[142]

Although the immobilization of biorecognition elements on the surface of the biosensing platform is a very important step for the design of sensitive, selective and long operational lifetime biosensors, it is clear that each method has several advantages and disadvantages. Various factors such as the immobilization matrix and the charge or functional groups of the biorecognition units guide the selection of the appropriate method, and thus, effective interfaces are created.

## 6. Label-Free Electrochemical Cancer Biosensors for Point-of-Care Applications

Label-free electrochemical biosensors have a high capability of being adapted into point-of-care (POC) systems that can be used for outside the laboratory testing to minimize the need for healthcare services such as hospitals [14,143,144,145]. In POC testing particularly, microfluidic devices have attracted great attention lately for effective and accurate cancer diagnosis owing to their ability to separate analytes at a good resolution in a rapid reaction time and to minimize the handling errors and costs [143]. As a result, promising detection systems with high performances are acquired with the elimination of the need for trained personnel. Recently, in the study by Keyvani et al., a POC sensing device for the detection of cervical cancer was developed for whole blood. This system identified cancer circulating DNA with high purity by the help of a graphene oxide-dependent electrochemical sensor platform by using differential pulse voltammetry [146]. In another study, Ming et al. fabricated a cellulose-paper-based POC testing with the modification of amino redox graphene, thionine, streptavidin integrated gold nanoparticles and chitosan for the detection of biomarker 17β-estradiol, which may be associated with breast cancer. The detection strategy, realized with differential pulse voltammetry in phosphate buffer solution, was carried out via the interaction of the target biomarker and its biotin-modified aptamer on the surface of the paper. The linearity of the label-free sensor was between 10 pg mL^−1^ and 100 ng mL^−1^, with a limit of detection value of 10 pg mL^−1^ [147].

Besides microfluidic devices, multiplex systems that can detect multiple analytes associated with cancer have several advantages in terms of label-free point-of-care testing. As an example, Kuntamung and his colleagues achieved simultaneous detection of breast cancer biomarkers: mucin1, cancer antigen 15-3 and human epidermal growth factor receptor 2 depending on the formed antibody and antigen interactions. For this purpose, redox species and antibody-conjugated polyethylenimine-modified gold nanoparticles were utilized as the modification elements of a SPCE. In addition to multiplex detection performance, the label-free biosensor kept 90% of its initial responses obtained via voltammetry [92]. In another approach that contained the fabrication of a flexible screen-printed electrode system, carcinoembryonic antigen was detected on graphene–ZnO nanorods deposited on a polyethylene terephthalate substrate with a screen-printed electrode by Chakraborty et al. ZnO nanorods were functionalized with aptamers and the resulting surface improved the mass transport through the electric field application. This system was integrated into smartphone interface technology and a handheld potentiostat. The linearity of the label-free sensor was between 0.001 pg mL^−1^ and 10 pg mL^−1^, with a limit of the detection value of 1 fg mL^−1^ by using electrochemical impedance spectroscopy. The results were also validated using a commercial ELISA kit [148].

The use of label-free POC testing in cancer diagnosis is in increasing demand in recent years since POC systems yield rapid decisions, more frequent testing to monitor wellness, eliminate the need for trained staff and utilize small specimen volumes. In addition, they are cost-effective. Despite these advantages, they are still more open to false positives or negatives and incorrect interpretations. Also, these sensing platforms have a risk of external interference since the environment is not as well controlled as in laboratories. In some cases, the sampling procedure can be inconvenient, such as in cancer diagnosis protocols. Indeed, POC-based electrochemical cancer biosensors are not yet available on the market. One of the additional reasons for this issue could be the distance between physicians and electrochemical biosensor developers. It is believed that multidisciplinary studies between them will improve the quality of the developed platforms. Additionally, shelf-life and production control are important parameters to improve their commercialization capacity [149,150,151,152]. However, electrochemically based POC systems are promising tools for the accurate and fast detection of cancer with their overall characteristics.

## 7. Conclusions and Future Perspectives

In the current review, we have summarized the recent achievements and progresses around label-free electrochemical biosensors that are utilized for cancer detection. Since the type of biorecognition element is an important key parameter to enhance the selectivity of the detection, the classification of the biosensors is made according to the types of recognition elements. Besides the achievements, the current challenges are also outlined in detail. Label-free detection systems are in urgent demand owing to their properties, including reducing labored modification steps and interference effects.

The growing demand on clinical research and the medical industry for cancer studies has pushed scientists to perform early detection with practical analytical tools instead of time-consuming and back-breaking methods. In addition to detection, isolation of the cancer cells is also important to increase the survival rates and quality of life. The design and development of early-cancer diagnosis platforms has been one of the hot topics of the last decades. The recent advances in the field of cancer diagnosis show that electrochemical sensing methodologies have an important impact on the accurate, rapid and sensitive detection of cancer types. Particularly, label-free electrochemical biosensors maintain predominant features to obtain reliable, cost-effective and selective cancer diagnosis that can serve for future implementations. With the addition of advanced materials such as nanomaterials, not only sensitivity of the biosensors but also the selectivity of them can be significantly improved. Surface modification makes bare electrode substrates available and suitable for biorecognition element immobilization. Recent studies on label-free and electrochemical biosensing of cancers indicate how promising and operational these biosensors are. It is certain that their advantages will certify more powerful medical applications in the near future with the support of growing materials science technology.

## Data Availability

Not applicable.
